# Costochondral junction variations in children younger than 2 years

**DOI:** 10.1007/s00247-025-06316-0

**Published:** 2025-07-17

**Authors:** Boaz Karmazyn, Matthew M. Jones, Lisa R. Delaney, Ralph A. Hicks, Ann E. Freshour, Megan B. Marine, Shannon L. Thompson, S. Gregory Jennings, George J. Eckert, Monica M. Forbes-Amrhein

**Affiliations:** 1 Riley Hospital for Children at IU Health, Department of Radiology and Imaging Sciences, Indiana University School of Medicine, 705 Riley Hospital Drive, Indianapolis, IN 46202 USA; 2 Department of Radiology and Imaging Sciences, Indiana University School of Medicine, Indianapolis, USA; 3 Riley Hospital for Children at IU Health, Department of Pediatrics, Section of Child Protection Programs, Indianapolis, USA; 4 Department of Biostatistics and Health Data Science, Indiana University School of Medicine, Indianapolis, USA

**Keywords:** Child abuse, Children, Computed tomography, Infant, Rib fractures

## Abstract

**Background:**

Costochondral junction fractures are considered specific for child abuse and typically heal with a deformed costochondral junction.

**Objective:**

To evaluate the types, location, and incidence of costochondral junction variations that can mimic fractures.

**Materials and methods:**

A 15-year retrospective study was conducted on children under 2 years of age who underwent chest and abdominal computerized tomography (CT) scans for pneumonia, fever, congenital lung disease, pain, or appendicitis. Randomized selection included 120 chest and 120 abdominal CT scans. Demographic and clinical information was obtained from medical record reviews. Two pediatric radiologists independently reviewed the studies and indicated the presence and location of costochondral junction variation patterns (spurs), and fissure, horizontal lucency, corner, or bucket handle as identified on two consecutive slices on axial views. Disagreements were resolved by a third radiologist. We excluded patients with underlying medical conditions that could affect the skeleton and studies with motion artifacts. A *t*-test was used to evaluate the relationships between age, CT slice thickness, and the diagnosis of costochondral junction variations. Kappa statistics were used to evaluate agreement.

**Results:**

A total of 123 children were excluded due to motion artifacts (*n* = 30), trauma (*n* = 31), being evaluated for child abuse (*n* = 3), slice thickness of 5 mm (*n* = 1), and underlying medical conditions (*n* = 58). The final group included 117 children (73 males and 44 females) with an average age of 1 year; 64 had chest and 53 abdominal CT scans. Agreement was fair (kappa = 0.29) at the patient level and poor at the rib level (kappa = 0–0.64). The final number of variations, after resolving disagreements with a third radiologist, was 46 of costochondral junction variations in 19 children (16.2%, 19/117); all were costochondral junction spurs at the levels of the second to eighth ribs. Costochondral junction variations were significantly more common in younger children (average 0.7 ± 0.6 years vs. 1.1 ± 0.6 years, *P* = 0.024) and when there was thinner CT slice thickness (average 1.6 ± 1.4 mm vs. 2.5 ± 1.5 mm, *P* = 0.041).

**Conclusion:**

Costochondral junction variations were identified in 16.2% of children under 2 years of age, and some may mimic healing costochondral junction fractures. There was only fair agreement between radiologists.

**Graphical abstract:**

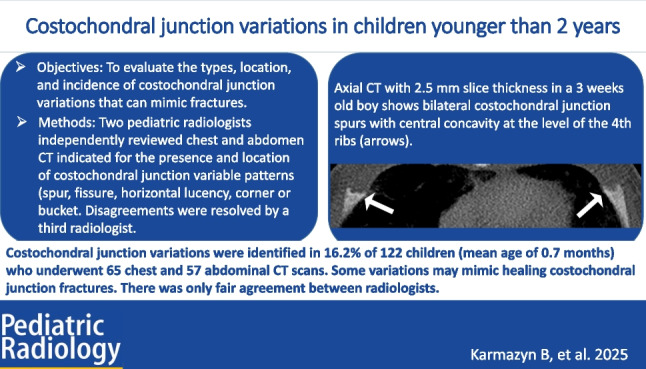

## Introduction

Rib fractures are among the most common fractures detected in children suspected of child abuse, with an incidence of 10% to 45.5% [1–5]. Rib fractures are also the most common fractures detected on postmortem radiographs of abused infants [6]. Posterior rib fractures in infants are highly specific injuries in child abuse [7].

Another type of rib fracture specific to child abuse, yet uncommonly seen, is the costochondral rib fracture [8]. These fractures are unique because they occur at the junction between the bony and cartilaginous portions of the rib, resulting in a different appearance compared to other types of rib fractures. Additionally, the healing process of this type of rib fracture differs, as subperiosteal new bone formation and callus are uncommon, with the most common sign of healing being growth disturbance of the costochondral junction [9]. Lastly, most costochondral junction fractures are noted to be in a healing phase at the time of initial diagnosis [9].

Costochondral junction fractures are best detected on chest computerized tomography (CT) and uncommonly detected on radiographs [10]. According to the ACR appropriateness criteria, chest CT may be appropriate when there is a high suspicion for abuse and the initial skeletal survey is negative [11]. With increased awareness of the specificity of costochondral junction fractures and the growing use of chest CT in our practice, we have encountered cases with low suspicion for child abuse in which the probable diagnosis of costochondral junction fractures on radiographs prompted a chest CT scan that showed costochondral junction deformities that mimicked healed costochondral junction fractures. This observation led us to question whether there are variations in the costochondral junction in children less than 2 years of age that can mimic a costochondral junction fracture.

## Material and methods

This study complied with the Health Insurance Portability and Accountability Act and was approved by our institutional review board with a waiver of informed consent. This was a 15-year retrospective study (2008–2022) on children younger than 2 years. Utilizing our institution’s radiology information system, we retrieved all chest and abdomen CT scans for children evaluated for pneumonia, fever, congenital lung disease, pain, or appendicitis. The scans were divided into eight age groups, in increments of 3 months. Within each age group, following randomization, 15 chest and 15 abdomen scans were included. Demographic and clinical information were obtained from medical record review.

**Fig. 1:**
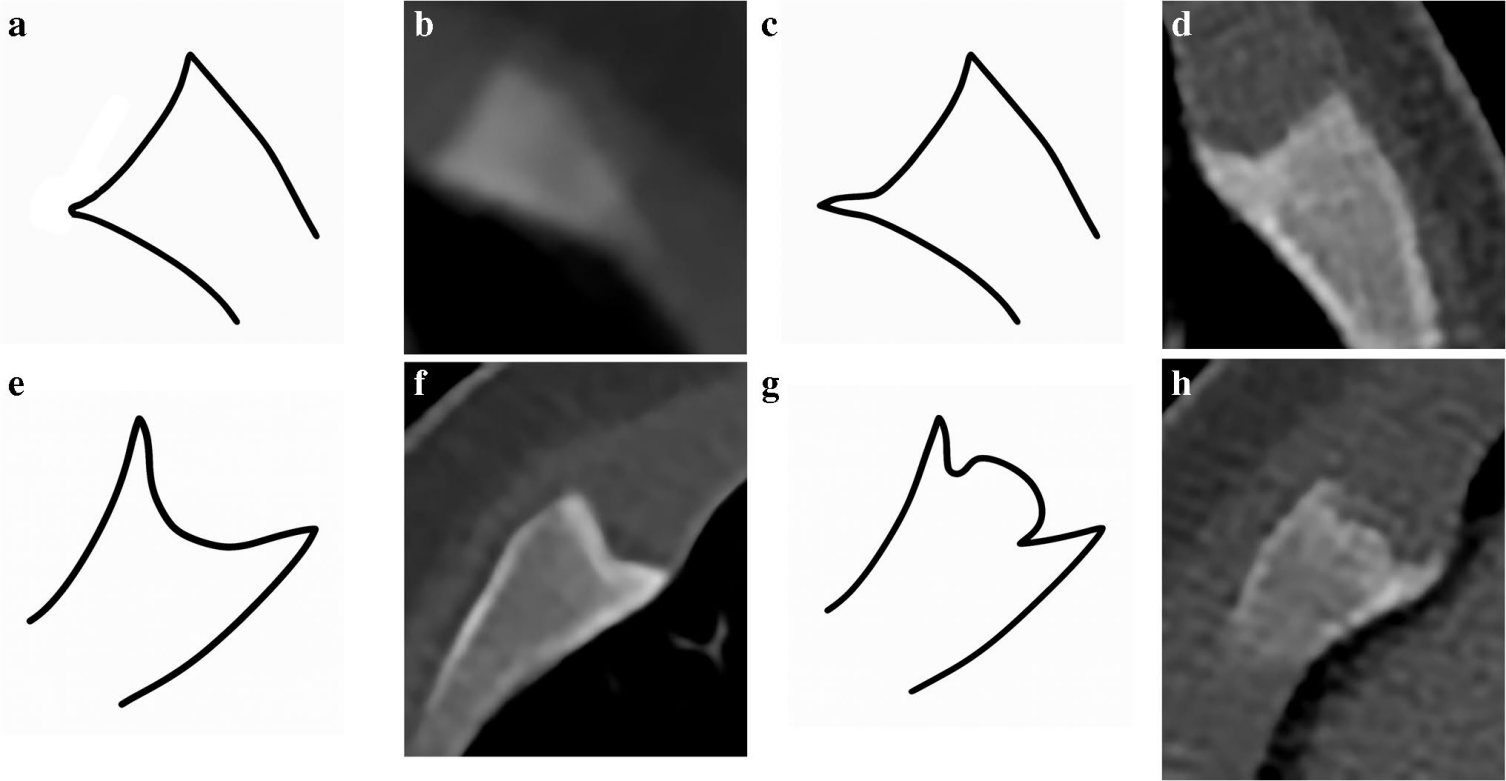
Types of costochondral spurs with flat, concave, and convex centers. **A** Illustration of the normal costochondral junction fracture that may be slightly concave. **B** Axial computerized tomography (CT) in a 3-month-old boy. Left 4th rib with minimal concave costochondral junction. **C** Illustration of a spur with a flat center. **D** Axial CT 2.5-mm slice thickness in a 3-month-old girl. Left 7th rib costochondral spur with a flat center. **E** Illustration of a spur with concave center. **F** Axial CT 2-mm slice thickness in a 3-month-old boy. Right 5th rib costochondral spur with a concave center. **G** Illustration of a spur with a convex center. **H** Axial CT 2.5-mm slice thickness in a 3-month-old boy. Right 7th rib costochondral spur with a convex center

The studies were anonymized and randomized. Two pediatric radiologists independently reviewed the studies and indicated the presence and location of the costochondral junction spurs (a beak-like projection) at the edge of the costochondral junction with three variations: flat, concave, and convex centers, as they can also mimic the growth disturbance of healing corner fractures (Fig. 1). In addition, the two radiologists evaluated for the presence of fissures, horizontal lucencies, and corner or bucket handle patterns as previously described by Tsai A. et al. representing patterns of acute costochondral junction fractures [9]. We additionally included spurs (a beak-like projection) at the edge of the costochondral junction with three variations: flat, concave, and convex centers, as they can also mimic healing corner fractures (Fig. 1).

Any disagreements were resolved by a third radiologist with 27 years of post-fellowship experience. When available, vitamin D levels were reviewed in children diagnosed with costochondral junction variations.

We excluded studies with slice thickness > 4 mm, motion artifacts that the radiologists reviewing the studies thought compromised evaluation of the costochondral junction, history of trauma, premature birth with gestational age < 34 weeks, bone dysplasia, chromosomal abnormalities, medical conditions and medications that can affect the bones, and cardiac arrest.

Mann–Whitney *U* tests were used to evaluate the relationships of age and CT slice thickness with diagnosis of costochondral junction variations. Kappa statistics were used to evaluate agreement. A kappa of 0.01–0.20 was considered none to slight, 0.21–0.40 fair, 0.41–0.60 moderate, 0.61–0.80 substantial, and 0.81–1.00 almost perfect agreement.

## Results

A total of 240 CT scans were identified. However, 123 children were excluded for the following reasons: motion artifacts (*n* = 30), trauma (*n* = 31), gestational age at birth < 34 weeks (*n* = 22), chromosomal abnormalities (*n* = 8), chemotherapy (*n* = 8), corticosteroid administration (*n* = 7), furosemide (*n* = 3), chest surgery (*n* = 3), being evaluated for child abuse (*n* = 3), Gaucher disease (*n* = 1), Alagille syndrome (*n* = 1), biliary atresia (*n* = 1), bone dysplasia (*n* = 1), carnitine palmitoyltransferase-2 deficiency (*n* = 1), rickets (*n* = 1), cardiac arrest (*n* = 1), and slice thickness of 5 mm (*n* = 1). None of the patients had undergone cardiopulmonary resuscitation.

**Fig. 2:**
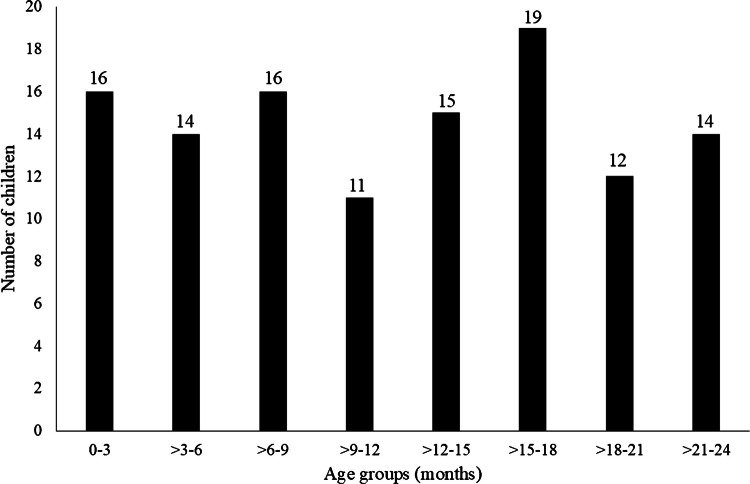
Distribution of children in 3-month age intervals in the final cohort of 117 children. The distribution of children per age group which ranged from 11 to 19 children (average 14.6 ± 2.3). At the *top* of each *column*, the *numbers* indicate the number of children

The final group included 117 children (73 males and 44 females) with an average age of 1 year, with 64 chest and 53 abdominal CT scans. Seven children had underlying medical conditions, including congenital heart disease (*n* = 1), myelomeningocele (*n* = 1), bladder exstrophy (*n* = 1), hydrocephalus (*n* = 1), polymicrogyria (*n* = 1), solitary kidney (*n* = 1), and nephrolithiasis (*n* = 1). Eight children were late preterm births (34 weeks to 36 weeks gestational age). Figure 2 shows the distribution of children per age group, which ranged from 11 to 19 children (average 14.6 ± 2.3).

As different protocols were used for different indications, the thinnest slice varied between studies. The slice thickness was ≤ 1 mm in 47 studies, 2–3 mm in 23 studies, and 3–4 mm in 47 studies. The average thinnest slice thickness was 2.3 mm.

The most common indications for chest CT were pneumonia 39.1% (25/64), evaluation for sequestration and/or congenital pulmonary airway malformation 37.5% (24/64), fever of unknown origin 9.4% (6/64), and foreign body aspiration 7.8% (5/64). The most common indications for abdomen CT were abdominal pain 60.4% (32/53), appendicitis 13.2% (7/53), and fever of unknown origin 13.2% (7/53).

Radiologists 1 and 2 identified 26 (22.2%, 26/117) and 20 (17.1%, 20/117) patients with costochondral junction variations, respectively. Costochondral junction spur was the most common variation. Radiologist 1 identified 52 costochondral junction spurs in 24 (20.5%, 24/117) patients and radiologist 2 identified 76 costochondral junction spurs in 18 (15.4%, 18/117) patients.

Two costochondral junction fissures in two patients were identified only by radiologist 1.

Costochondral junction corner (*n* = 3), costochondral junction bucket handle (*n* = 1), and costochondral junction horizontal (*n* = 1) variations were identified in three (2.6%, 3/117) patients by radiologist 1. Costochondral junction corner (*n* = 1) and horizontal (*n* = 1) variations were identified in two (1.7%, 2/117) patients by radiologist 2. Details of the types of costochondral junction variations are summarized in Table 1.

**Table 1:** Types of costochondral variations detected by radiologists 1 and 2 at the rib level

Variation patterns	Radiologist 1				Radiologist 2				Average kappa
	Sum	Right	Left	Bilateral	Sum	Right	Left	Bilateral	
Spur	52	28	18	3	76	30	18	14	0.02
Flat	26	13	7	3	16	7	3	3	0.02
Concave	9	5	4		47	15	12	10	0.09
Convex	17	10	7	0	13	8	3	1	0.30
Fissure	2				0				No agreement
Corner	3	3			1		1		No agreement
Bucket handle	1		1		0				No agreement
Horizontal	1	1			1	1			No agreement

There was fair agreement (kappa = 0.29) between radiologists on patients with costochondral junction variations and poor agreement (kappa ranging from 0 to 0.64, average 0.20) at the rib level. There was also poor agreement on the different types (flat, concave, convex) of costochondral junction spurs (kappa range 0 to 0.26 with average 0.02 for flat, 0 to 0.39 with average 0.09 for concave, 0 to 66 with average 0.30 for convex). There was no agreement between radiologists on the presence of costochondral junction fissure, corner, bucket handle, and horizontal variations, and none was observed after resolution of disagreement.

**Fig. 3:**
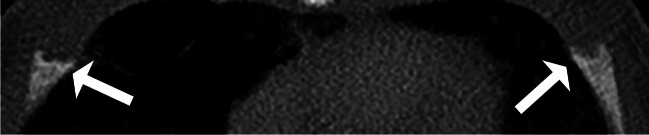
Symmetric bilateral costochondral junction spurs in a 3-week-old boy evaluated with chest computerized tomography (CT) due to respiratory distress. Axial CT with 2.5-mm slice thickness shows bilateral costochondral junction spurs with central concavity at the level of the 4th ribs (*arrows*)

**Fig. 4:**
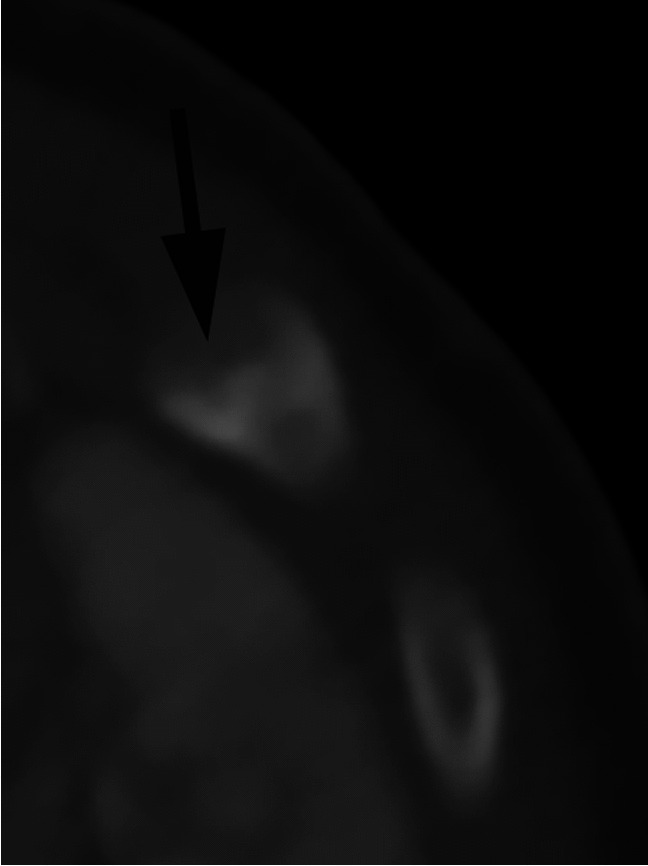
Asymmetric left costochondral junction variation in a 3-month-old boy evaluated for congenital pulmonary airway malformation. Axial computerized tomography with 4-mm slice thickness shows a left costochondral junction spur with central convexity (*arrow*)

**Fig. 5:**
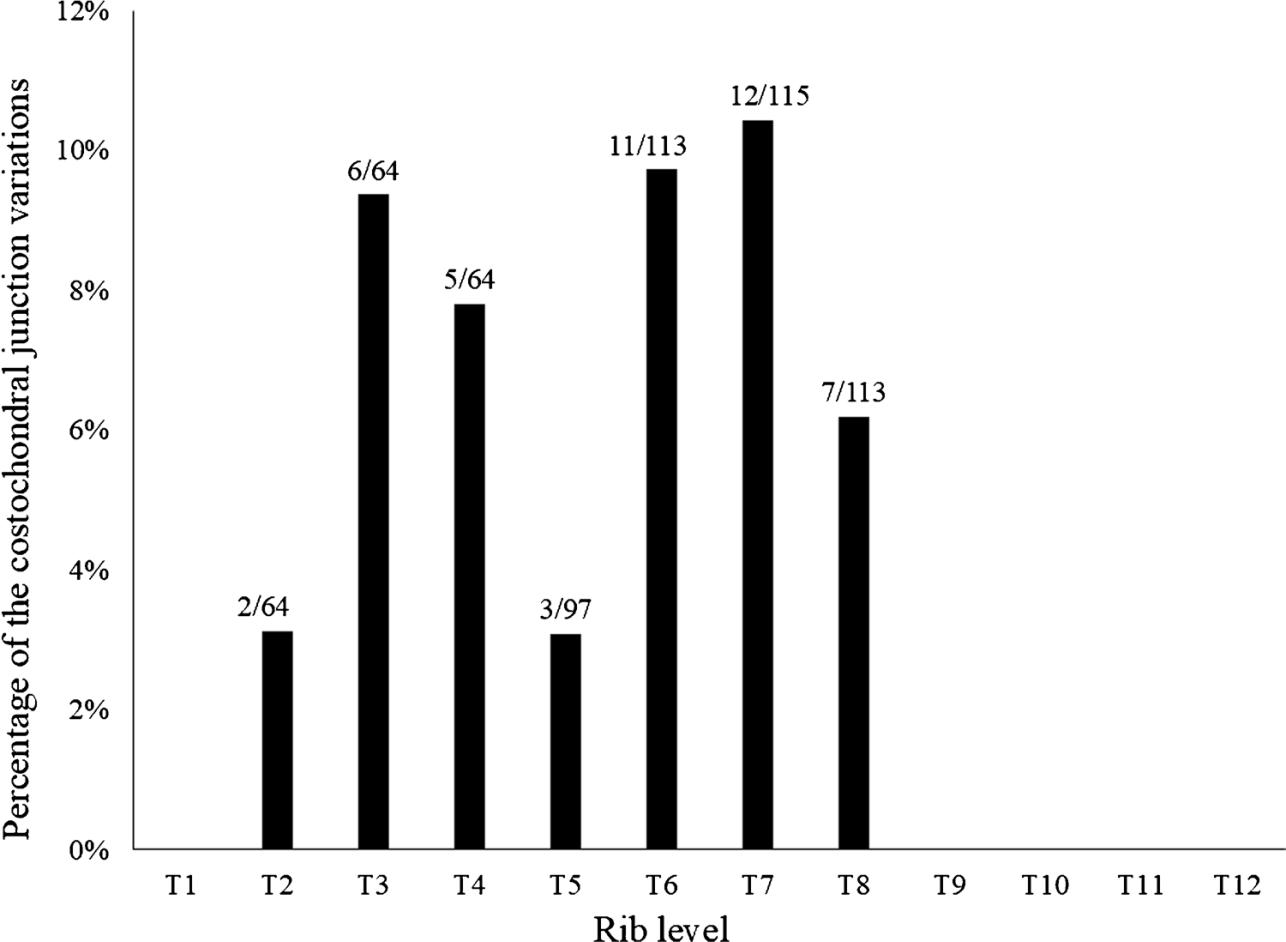
Percentage of ribs with costochondral junction variations after consensus read corresponding to specific rib levels. At the *top* of each *column*, the *numerator* indicates the number of costochondral junction variations, and the *denominator* represents the number of ribs evaluated. All variations were costochondral junction spurs

The two radiologists agreed on the presence of 15 costochondral junction variations in eight patients. The final number of children with variations after resolution of disagreements by a third radiologist was 19 children (16.2%, 19/117) who had 46 costochondral junction variations. Seventeen costochondral junction variations (37.0%, 17/46) were only on the right, nine (19.5%, 9/46) only on the left, and 20 (43.5%, 20/46) were bilateral at the same rib level. All of these were costochondral junction spurs (Figs. 3 and 4). Costochondral junction spurs were observed from the second to the eighth rib levels (Fig. 5). Vitamin D levels were available in only two of the 19 children with a final diagnosis of costochondral junction variation, and both values were within normal limits.

Costochondral junction variations were significantly more common in younger children (average 0.7 ± 0.6 years vs. 1.1 ± 0.6 years, *P* = 0.024) and when there was thinner CT slice thickness (average 1.6 ± 1.4 mm vs. 2.5 ± 1.5 mm, *P* = 0.041).

## Discussion

Costochondral junction variations were found in children with an average age of 0.7 years (8.4 months), which is an age when children are vulnerable to child abuse [12]. In our series, a costochondral junction variation was demonstrated at the level of the second to the eighth ribs in 16.2% of the CT scans, and only 43.5% were symmetric at the same level. The only type of costochondral junction variation identified after disagreement resolution was a costochondral junction spur.

Costochondral junction fractures are uncommonly detected on radiographs when compared with chest CT [10]. For this reason, our study focused on CT scans rather than on radiographs. We included both chest and abdominal CT scans to include all of the ribs.

Costochondral junction fractures are unique types of rib fractures, as the costochondral junction is analogous to the metaphysis of the long bones in extremities. Histological studies of the costochondral junction during late intrauterine development and the neonatal period have demonstrated well-organized zones that mirror the architecture of the growth plate in long bones. The cartilage–ossification junction is typically demarcated by a well-defined, often curved line [13, 14]. Costochondral junction fractures have distinctive patterns both seen histologically [6] and on radiographs, with similarities to the classic metaphyseal lesions [8, 9, 15]. A study by Forbes-Amrhein et al. found that costochondral junction fractures were highly specific for child abuse. The rarity of this type of fracture, even in known high-energy accidental trauma, was noted in this study’s cohort of 22 young patients with 109 costochondral junction fractures [8].

Like the classic metaphyseal lesion (CML), the healing phase of costochondral junction fractures is unique in that subperiosteal new bone formation and callus are uncommonly found [9]. Growth disturbance is the most common finding of a healing costochondral junction fracture [9, 13], raising concern that normal costochondral junction variations may mimic this appearance. Costochondral junction fissure, corner, bucket handle, and horizontal patterns were described in Tsai et al.’s study. The acute phase of costochondral junction fractures with a bucket handle pattern was the most common type of acute costochondral junction rib fracture seen [9]. In our study, radiologists 1 and 2 identified these costochondral junction patterns in only a few cases (2.6% and 1.7%, respectively). There was no agreement between radiologists on any of these costochondral junction patterns.

Our study found only fair agreement (kappa = 0.29) for detection of costochondral junction variations. A prior study by Karmazyn et al. showed almost perfect and substantial agreement for positive chest CT scans and rib fractures (kappa of 0.82 and 0.63, respectively) [11]. This study included any type of rib fracture, rather than solely costochondral junction fractures. In addition, it is possible that the CT patterns of costochondral junction variations are milder and more challenging to detect as compared to costochondral junction fractures. The agreement for the subtypes of the costochondral junction spurs (flat, concave, and convex) was poor; and therefore, the use of different spur categories may not be of practical use. In our study, a review by a third radiologist eliminated the few cases with patterns that could mimic acute costochondral junction fractures.

Costochondral junction variations were significantly more common in younger children (average 0.7 ± 0.6 years) which is the period when children are more vulnerable for child abuse [12]. It is therefore important to be aware of the costochondral junction variations when evaluating rib fractures in infants.

Our study has several limitations. The study included children younger than 2 years, as skeletal surveys are routinely performed in this age group for the evaluation of suspected child abuse. However, the risk of abuse is highest in infants [12], making this the most critical age for accurately distinguishing normal variants from true fractures. A limitation related to age distribution was the variation in the number of children across age groups; however, this variation was relatively minor, with a standard deviation of only 2.3 children. Although none of the children with costochondral variation had a clinical diagnosis of rickets, and two had documented normal vitamin D levels, the possibility of subclinical vitamin D deficiency cannot be entirely excluded. However, this is considered unlikely, as all of the children exhibited sporadic, mostly non-symmetric variations rather than the diffuse, bilateral findings typically associated with rickets. Not all chest and abdominal CT scans were performed with the same protocol and slice thickness. This reflects the clinical practice in which the thinnest slice utilized depends on the indication of the study. Costochondral junction variations were detected in any slice thickness, but significantly more so with thinner CT slice thickness. This could bias detection of costochondral junction variations in the lower ribs, which were more often scanned by abdominal CT scans that used thicker CT slice thickness compared to chest CT. In addition, this study was based on axial images; 3D volume rendering of the ribs could have better demonstrated the anatomy of the costochondral junction and improved detection of variations [16]. As the radiologists focused on the evaluation of costochondral junction variations, there may have been some bias toward overdetection of these costochondral junction variations. Some of these junctions may have only minimal costochondral junction deformity and may be ignored when evaluating for costochondral junction fractures. In addition, although we selected a patient population with low risk for child abuse, this could not be entirely excluded in the absence of a gold standard.

In conclusion, radiologists should be aware of costochondral junction variations, some of which can potentially mimic healing costochondral junction fractures. Costochondral junction variations most often had a spur pattern and were identified in 16.2% of children younger than 2 years at the level of the second to eighth ribs. Costochondral junction variations were more commonly identified with thin-slice CT scans and in younger children (average age of 0.7 years or 8.4 months). Radiologists should be aware of the limitations in the diagnosis of costochondral junction variations, as there was only a fair agreement between radiologists.

## Data Availability

The data underlying this study are not publicly available in order to protect the privacy of study participants.
